# Performance of Human Gene EPB41L3 and HPV 16/18 Viral DNA Methylation to Triage hrHPV-Positive Women

**DOI:** 10.3390/vaccines12010046

**Published:** 2023-12-31

**Authors:** Remila Rezhake, Yan Wang, Xuelian Zhao, Marc Arbyn, Guqun Shen, Qinjing Pan, Xun Zhang, Yuanming Zhang, Fanghui Zhao, Youlin Qiao

**Affiliations:** 1 Cancer Research Institute, Affiliated Cancer Hospital of Xinjiang Medical University, Urumqi 830000, China; remila@xjmu.edu.cn (R.R.); xjwangyan2012@163.com (Y.W.); 13565938887@163.com (G.S.); qiaoy@cicams.ac.cn (Y.Q.); 2 Department of Cancer Epidemiology, National Cancer Center/National Clinical Research Center for Cancer/Cancer Hospital, Chinese Academy of Medical Sciences and Peking Union Medical College, Beijing 100021, China; xuelian503@126.com (X.Z.); pqjing@hotmail.com (Q.P.); zhangxun7508@163.com (X.Z.); 3 Unit of Cancer Epidemiology, Belgian Cancer Centre, Sciensano, Brussels B-1000, Belgium; marc.arbyn@sciensano.be; 4 School of Population Medicine and Public Health, Chinese Academy of Medical Sciences and Peking Union Medical College, Beijing 100021, China

**Keywords:** methylation, cervical cancer, HPV, cancer screening, early detection, triage, biomarker

## Abstract

More evidence from population-based cohort studies is required to confirm the application of methylation-based biomarkers in real-world settings. The cross-sectional and 24-month cumulative triage performance of a novel methylation assay targeting the host gene EPB41LE and HPV16/18 DNA L1/L2 regions among hrHPV-positive women was evaluated based on a population-based cohort study from China. Overall methylation positivity was 12.4% among hrHPV-positive women. Methylation-positive women had significantly higher risks of hrHPV persistence at 12M and 24M follow-up (RR_12M_ = 1.9, 95%CI: 1.5–2.6 and RR_24M_ = 1.7, 95%CI: 1.2–2.5). For CIN2+, cross-sectional triage sensitivity of methylation was similar to HPV16/18 (70.6% vs. 64.7%, p_exact_ = 1.000), but was lower than cytology (94.1%), although not significantly (p_exact_ = 0.213). The specificity (91.2%) of methylation was significantly higher than other triage methods (*p* < 0.001 for all). The longitudinal sensitivity of methylation over 24M follow-up was 56.0%, lower (but not significantly so) than HPV16/18 (64.0%, p_exact_ = 0.688) and cytology (76.0%, p_exact_ = 0.125). Methylation testing showed high positive predictive values for CIN2+ (41.4% at baseline, 50.0% at 24-month), while the CIN2+ risk of methylation negative women (cNPV) remained considerable (2.5% at baseline, 6.9% at 24-month). Study findings indicate that methylation has better specificity and predictive values for the presence or development of cervical precancer and might therefore be considered for the strategy of HPV screening and methylation triage followed by immediate treatment of triage-positive women and delayed follow-up of hrHPV-positive/methylation-negative women.

## 1. Introduction

The discovery of the etiological relationship between high-risk human papillomavirus (hrHPV) persistent infection and cervical cancer provides the ideal opportunity to prevent and ultimately to eliminate cervical cancer by HPV vaccination, screening, diagnosis and treatment of early detected lesions [1,2]. Nonetheless, cervical cancer remains the fourth most common cancer worldwide, with a remarkable high burden in low- and-middle income countries (LMICs) [3]. Considering the fact that vaccination is not yet available in many low- and middle-resource settings due to lack of funding and political support at the government level, lack of awareness among healthcare providers and the perceived cost/benefit ratio for the individual level, screening will remain as the main preventive strategy of cervical cancer for the adult women in coming decade, whereas HPV vaccination will have its main effect among future generations [4,5,6]. In its latest guidelines, the WHO recommended to use HPV testing as a primary screening method rather than visual inspection with acetic acid (VIA) or cytology [7]. Management of all HPV-positive women may result in considerable over-referral, over-diagnosis and over-treatment. Thus, accurate triage methods identifying women at high risk of persistent infection and high-grade lesions are urgently required to increase screening efficiency.

Currently, cytology and HPV16/18 genotyping are the most commonly used triage methods, but due to the subjective characteristics of cytology and type restriction of genotyping, appropriate triage strategies still need to be determined. DNA methylation has been shown as a new and promising triage option for hrHPV-positive women [8,9,10]. Various methylation methods targeting different genes of host or/and HPV have been explored [11]. In a recent meta-analysis, DNA methylation, used to triage hrHPV-positive women, showed a significantly higher specificity than cytology at cut-off ASCUS+ and higher sensitivity than HPV16/18 genotyping [10]. Despite the increasing number of methylation studies, more longitudinal studies in population settings are needed to increase the strength of the current evidence.

With the present study, we aimed to evaluate the cross-sectional and longitudinal performance (over 24 months (M)) of methylation biomarkers targeting the human EPB4IL3 gene and the HPV 16/18 L1/L2 genes among hrHPV-positive women in comparison with cytology and genotyping in a population-based screening study from a rural area of northwest China.

## 2. Materials and Methods

### 2.1. Study Design and Baseline Screening Procedure

**Figure 1:**
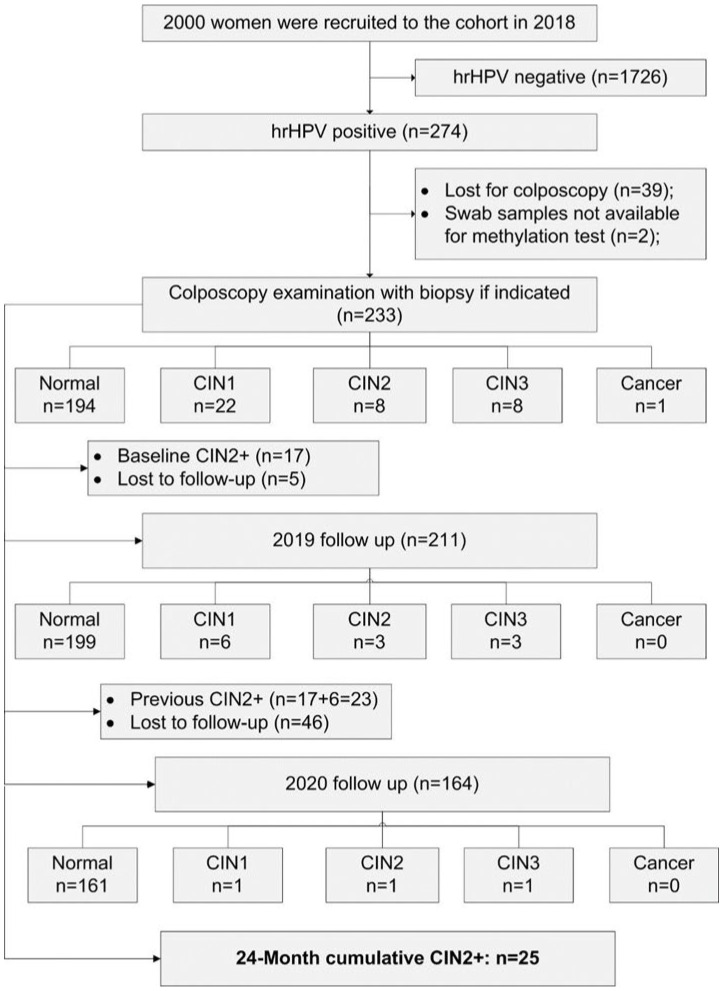
Study flowchart showing baseline and 24-month follow-up endpoints. hrHPV, high-risk human papillomavirus; CIN1-3, cervical intraepithelial neoplasia grade 1–3; CC, cervical cancer.

Between June to August in 2018, a total of 2000 women aged 25–64 from Tuoli County, Xinjiang, China, were recruited to this prospective cervical cancer screening cohort study. Details on the study design and baseline information have been previously published [12]. In brief, women who were not pregnant, had not been treated for cervical intraepithelial neoplasia (CIN) in the last 5 years and who consented to participate in the study were eligible for inclusion to the cohort. Exclusion criteria were pregnancy, previous total hysterectomy and inability to comply with the study protocol. For the current analysis, only women who (i) were hrHPV-positive and had attended a colposcopy examination at baseline, (ii) had baseline swab samples available, (iii) and had been followed up until 2020 were included (Figure 1). The study was approved by the Ethical Committee of The Affiliated Cancer Hospital of Xinjiang Medical University, China (Approval number: K-201802).

Informed consent was obtained by local healthcare personnel after explaining the study procedure. All women were invited to answer a questionnaire regarding demographic, gynecologic and obstetric history, then underwent a pelvic examination followed by collection of cervical samples by a gynecologist in the following order: a cervical swab (dacron) sample; a second cervical cytology sample collected with sample collection brush and placed in Sample Preservation Solution (Shenzhen Senying Biotechnology Co. Ltd., Shenzhen, China); a third sample collected using the careHPV collection device and placed in specimen transport medium (Qiagen, Shenzhen, China). All laboratory tests were performed totally blinded to the other screening results.

### 2.2. Laboratory Tests

careHPV test: careHPV is a nucleic acid hybridization assay with signal amplification using microplate chemiluminescence for the detection of 14 high-risk HPV types (16, 18, 31, 33, 35, 39, 45, 51, 52, 56, 58, 59, 66, 68) in bulk. careHPV testing was carried out at the HPV laboratory of Tuoli Maternal and Child Health Hospital according to the standard protocol. Samples were considered hrHPV-positive if relative light units (RLU/CO) were ≥1.0.

GenPlex^®^ HPV test: GenPlex^®^ HPV test (Human Papillomavirus Genotyping Kit (Microfluidic Chip), BOHUI, Beijing, China) is a multiplex Polymerase Chain Reaction (PCR) test. Type-specific primers target the L1 region of the HPV genome, whereas identification of amplicons is performed by reverse DNA hybridization using DNA chip technology. The test separately detects 24 HPV genotypes, including HPV6, 11, 16, 18, 31, 33, 35, 39, 42, 43, 44, 45, 51, 52, 53, 56, 58, 59, 66, 68, 73, 81, 82 and 83. In the present study, GenPlex^®^ HPV testing was performed according to the manufacturer’s instructions using 0.5 mL from the third cervical specimen remnant after careHPV testing.

*Cytology:* The cytology specimen in the Sample Preservation Solution was used to prepare a slide for liquid-based cytology (LBC) using the Papanicolaou staining method. Results were interpreted according to the Bethesda 2014 classification system by experienced cytology technicians blinded to other screening results [13]. Atypical squamous cells of undetermined significance or worse (ASC-US+) were considered abnormal. All cytology slides were double read; namely, they were examined by a cytologist from the Affiliated Cancer Hospital of Xinjiang Medical University and re-reviewed by a senior expert from Cancer Hospital/Chinese Academy of Medical Sciences (CHCAMS).

*Methylation:* The swab samples were collected at baseline in 2018 and stored at −80 °C until being tested for methylation in 2020. careME methylation test (careLYFE, Suzhou, China) was used, which is based on methylation-specific real-time PCR techniques and targets the host cell gene EPB41L3 and viral HPV16L1/HPV18L2 genes. Firstly, the swab sample was vortexed for 2 min in 600 µL lysis buffer, then used for DNA extraction by the Magnetic DNA Puri Kit (careLYFE, China) according to the manufacturer’s instructions. The 40 µL eluted and purified DNA was used for the bisulfite conversion reactions where unmethylated cytosine was converted to uracil, then the converted DNA was used for desulphonation and clean-up with the Magnetic DNA methylation kit (careLYFE, China). careME methylation assay was based on 2 tubes of methylation-specific multiplex real-time PCR. A pair of methylation-specific EPB41L3 primers/probe covering targeted CpG positions were used for EPB41L3 CpG detection. Another pair of methylation-nonspecific ACTB primers/probe were used as internal control for total bisulfite conversion to normalize the methylation level of EPB41L3 precisely. For the 2-plex EPB41L3 PCR reaction, different fluorescent signals labeled in different probes were used for different gene testing. Similarly, a 4-plex HPV16L1&HPV18L2 methylation assay was established containing 4 pairs of primers/probe, one specific for HPV16L1 methylation and one for the internal control of HPV16, another one specific for HPV18L2 methylation and one for the internal control of HPV18. Briefly, for 2-plex EPB41L3 PCR, 10 µL of PCR master mix, 5 µL of converted DNA, 1 µL of primer (0.4 µmol/L of each primer), 1 µL of probe (0.2 µmol/L of each probe), 0.2 µL HotStar Taq DNA polymerase (1U) were used; for 4-plex HPV16L1&HPV18L2 PCR, 10 µL of PCR master mix, 5 µL of converted DNA, 1 µL of primer (0.4 µmol/L of each primer), 1 µL of probe (0.2 µmol/L of each probe), 0.25 µL HotStar Taq DNA polymerase (1.25U) were used; both reactions were adjusted with water to give a final 25 µL reaction volume and run at thermal cycling conditions initiated at 94 °C for 10 min, followed by 45 cycles: 20 s at 94 °C, 45 s at 62 °C, then final 10 s at 12 °C for hold. Signals were collected in the stage of 45 s at 62 °C. From DNA extraction to methylation-specific PCR, high-methylation positive control (PC), non-methylation negative control (NC) and non-template blank control (BC) were tested in each run in parallel. Total running time of the test is about 4 h including DNA extraction (~0.5 h), transformation of bisulfite (~2 h), amplification and detection (~1.5 h). According to the result of EPB41L3 and HPV16L1/HPV18L2 PCR assays, a Risk value (R value) for each sample was calculated by the Ct value of each gene and then normalized by each internal control gene. The final R value was calculated using secondary logistic probability regression for the combination of EPB41L3 and HPV-related gene methylation markers in CIN1-/CIN2+ in previous validation studies in laboratory. A cutoff Risk value of ≥1.8 derived in a previous cutoff study was regarded as a positive methylation result, indicating a high risk of high-grade cervical lesions and cervical cancer, which means further referral for colposcopy or other follow-up tests are required. R value < 1.8 indicates that the result is regarded as methylation negative, indicating a low risk of high-grade cervical disease and no need for referral to colposcopy.

### 2.3. Outcome Verification

Women positive for one or more of the 14 hrHPV types by either HPV test and/or with a cytology result of ASCUS+ were referred to colposcopy examination. Colposcopy was performed according to accepted diagnostic standards [14]. Biopsies were taken if clinically indicated. Cases with negative colposcopic impression where no biopsies were taken were considered negative for disease. Biopsy-confirmed CIN2+ were used as clinical outcome endpoints. All histology slides were reviewed by an experienced pathologist from the Affiliated Cancer Hospital of Xinjiang Medical University and re-confirmed by the pathologist from CHCAMS.

### 2.4. Follow-Up Procedure

All women with either hrHPV-positive or cytology ASC-US+ results, except those histologically confirmed CIN2+ at baseline, were called back for annual follow-up in 2019 and 2020. HrHPV and cytology tests were used in follow-up visits and any positive results were referred to colposcopy examination with biopsy if necessary. Women who were hrHPV-positive but did not attend the annual follow-up or failed to come back for the colposcopy examination were regarded as lost for follow-up. All laboratory tests and clinical diagnosis were conducted as described in the previous text.

### 2.5. Statistical Analysis

Pearson chi-square test was used to assess differences in methylation positivity rates by population characteristics. Chi-square test for trend was used to assess whether methylation test positivity increased by severity of the cervical lesions and respective odds ratios (ORs) with 95% confidence intervals (CIs) were calculated. Twelve-month (12M) and 24-month (24M) risk of hrHPV persistence was evaluated by risk ratio (RR) with respective 95% CIs. Sensitivity, specificity, positive predictive value (PPV) and negative predictive value (NPV) with respective 95% CIs for detecting CIN2+ were calculated for different triage strategies. A difference in sensitivity and specificity between given triage strategies and the reference triage was considered significant if the 95% CIs around the relative sensitivity and specificity did not include 1. We also calculated the referral rates (based on % triage test positivity) and the number needed to refer to colposcopy to find one case of CIN2+ (NNR) to evaluate the triage efficiency. All statistical tests were two-sided using a 0.05 significance level. Stata (version 12.0, StataCorp, College Station, TX, USA) was used for statistical analyses.

## 3. Results

### 3.1. Description of the Study Cohort

Figure 1 demonstrates the baseline and follow-up findings of the cohort. Overall, 2000 eligible women aged 25–64 years, with a median age of 40 years (interquartile range (IQR) 34–46 years), were recruited to this study. At baseline screening, 274 women (13.7%) were hrHPV-positive and were referred to colposcopy with a completion rate of 85% (233/274). Of these (n = 233), 17 women had histology confirmed CIN2+, 22 had CIN1 and 194 did not have CIN lesions. Over the whole 24M follow-up period, a total of 25 CIN2+ cases were detected.

### 3.2. Demographic and Clinical Characteristics of Study Population at Baseline

Characteristics of study population, positivity of methylation and respective odds ratio for each group are presented in Table 1. Methylation positivity was significantly higher in women older than 40 years with an odds ratio of 3.99 (95%CI: 1.56–10.20). Only 14% of women reported having had sex before 18 years old and their risk of having positive methylation results was not significantly higher than women who first had sex at later age (OR = 2.18, 95%CI: 0.85–5.60). Both pre- and post-menopausal women had a significantly lower risk to be methylation-positive compared to peri-menopausal women with ORs of 0.15 (95%CI: 0.04–0.52) and 0.23 (95%CI: 0.06–0.88), respectively. Women with HPV16/18 infection had 19-times higher risk of being methylation-positive (OR = 19.17, 95%CI: 7.26–50.61). Methylation positivity increased with the severity of cytology and histology grades (*p* < 0.001 by Chi square test for trend).

### 3.3. Clinical Performance of Different Triage Tests According to Baseline Findings

Table 2 shows the performance of different triage strategies among hrHPV-positive women according to baseline findings. The overall test positivity (colposcopy referral rates) of methylation was 12.4%, which was significantly lower than those of cytology (ASC-US+) triage (26.1%, *p* < 0.001) and HPV16/18 triage (24.5%, *p* < 0.001). Methylation showed lower sensitivity compared to cytology triage at ASC-US cut-off, although this was not statistically significant due to the limited CIN2+ cases (70.6% vs. 94.1%, relative sensitivity at 0.75, 95%CI: 0.53–1.06), while the specificity of methylation was significantly higher (92.1% vs. 79.2%, relative specificity at 1.16, 95%CI: 1.08–1.25). Compared to HPV16/18 genotyping, methylation showed slightly higher sensitivity but the difference was again not significant (70.6% vs. 64.7%, relative sensitivity at 1.09, 95%CI: 0.81–1.47); however, the specificity was significantly higher (92.1% vs. 78.7%, relative specificity 1.17, 95%CI: 1.10–1.25). Among the evaluated triage strategies, methylation showed the highest PPV at 41.4% with the lowest number of colposcopy referrals to detect one CIN2+ case (NNR = 2.4). The cNPV (1-NPV) of methylation was 2.5%, considerably higher than cytology ASC-US+ (0.6%), but lower than that of HPV16/18 triage (3.4%). The combined triage strategy of HPV16/18 with reflex methylation detected one more CIN2+ case than methylation alone at the cost of doubling the colposcopy referral rate and leading to a large loss in specificity and PPV.

### 3.4. Long-Term Risk Prediction Value of Methylation

As Table 3 shows, women who tested methylation-positive at baseline had significantly higher risk of having a persistent hrHPV infection at 12M follow-up and 24M follow-up with the respective RR values of 1.89 (95%CI: 1.42–2.52) and 1.65 (95%CI: 1.11–2.45), which were higher than the RR associated with baseline HPV16/18+ and cytology (ASC-US+) results. Moreover, methylation-positive women had a 7-times higher risk of having CIN2+ at 24M compared to methylation-negative women (RR = 7.27, 95%CI: 3.68–14.36). The highest risk of CIN2+ was associated with ASC-US+ cytology (RR = 8.98, 95% CI: 3.81–21.19) among single triage strategies. The longitudinal sensitivity of methylation for CIN2+ during 24M was 56.0% (95%CI: 34.9–75.6), which was lower than cytology (ASC-US+) and HPV 16/18 triage algorithms (76.0% and 64.0%), although both differences did not show statistical significance due to the limited CIN2+ cases in the cohort. The longitudinal specificity (91.4%, 85.9–95.2) and PPV (50.0%, 95%CI: 30.6–69.4) of methylation was significantly higher than either cytology or HPV16/18 genotyping. Very similar triage performance was found between methylation and cytology (LSIL+) algorithms. Of note, the risk of CIN2+ among methylation-negative women (cNPV = 1-NPV) was the highest (6.9%) at 24M follow-up among all evaluated triage strategies.

## 4. Discussion

In recent years, several biomarkers such as p16/Ki67, E6/E7 oncoprotein and methylation of viral or human genes have shown promising results in triaging hrHPV-positive women. However, more evidence regarding comprehensive screening performance and predictive value of such biomarkers in population-based programs, especially from the longitudinal perspectives, is required. In our study, we evaluated a novel methylation test targeting the human gene EPB41L3 and viral HPV16L1/HPV18L2 genes as potential markers to triage hrHPV-positive women in a population-based cohort study with 24 months of follow-up. Our results showed that the EPB41L3 and HPV16/18 methylation test has a significant advantage in improving triage specificity compared to HPV16/18 and cytology algorithms. Regarding triage sensitivity, although our data indicated only a slightly higher sensitivity of methylation compared to HPV16/18 triage and a lower sensitivity than cytology triage, these differences did not reach statistical significance mainly due to the limited number of CIN2+ cases detected at baseline and in the follow-up period. The overall pattern of triage performance of the methylation assay used in our study was similar to that from a recent meta-analysis, where the relative sensitivity of methylation for CIN2+ among hrHPV+ women was 0.81 (95%CI: 0.63–1.04) compared to cytology (ASCUS+) and 1.22 (95%CI: 1.05–1.42) compared to HPV16/18 genotyping, while the relative specificity was 1.25 (95% CI: 0.99–1.59) and 1.03 (95%CI: 0.94–1.13), respectively [10].

Various candidate genes, targeting either host or HPV viral genomes, have been studied in recent years as the targets of methylation sites [15,16,17,18]. Vasiljević et al. studied the methylation of 26 genes and concluded that EPB41L3 is one of the best human methylation genes that is clinically appropriate for triage of hrHPV-positive women [15]. Furthermore, the elevated methylation of the HPV L1 and L2 open reading frames is particularly associated with CIN2, CIN3 and invasive cancer, while the methylation of CpG sites in the URR, E6 and E7 regions of the HPV types is low and most differences are not significant [11,19,20,21]. Among different panels of methylation, a combination of methylation panels targeting the human gene EPB41L3 and the most carcinogenic HPV types, i.e., HPV16/18/31/33 (named S5 classifier), has been one of the most studied methylation tests among different populations, such as cancer patients [22], HPV-positive women with mild cytology abnormalities [23,24], colposcopy referral population due to HPV16/18+ and/or cytology abnormalities [25], or selected hrHPV+ women derived from a population-based screening program [26,27], even in HIV women [28]. All mentioned studies exhibited desirable accuracy and feasibility of the S5 classifier test, but population-based cohort studies are still lacking. Given the fact that HPV16/18 are the most carcinogenic types of HPV worldwide, and in order to improve the specificity of triage, the careME methylation assay, a relatively simple qPCR commercial version of the S5 classifier, was developed only targeting the human gene EPB41L3 and viral genes HPV16L1 and HPV18L2. Technically, careME is expected to cost a third less than S5 and uses a fluorescent PCR endpoint instead of pyrosequencing [25]. To our knowledge, our study provides the first evidence regarding the feasibility and clinical accuracy of this novel assay. Overall, the careME assay in our study showed slightly decreased sensitivity as compared to S5 in hrHPV+ women reported by other studies, but the specificity was remarkably higher [26,27].

Longitudinal data regarding hrHPV persistence and risk of high-grade cervical lesions offered by different methylation results are lacking. With the advantages of intensive follow-up of hrHPV-positive women in this study, we further explored the potential of methylation testing in predicting hrHPV persistence and CIN2+ occurrence. The results showed that methylation of EPB41L3, HPV16L1 and HPV18L2 predicted nearly two-times higher risk of hrHPV persistence at 12M and at 24M, demonstrating higher positive prediction values than HPV16/18 and cytology. Flatley et al. [29] reported that methylation of the tumor suppressor gene DAPK was associated with a 2.64-fold (95%CI, 1.35–5.17) increased risk of persistent hrHPV infection, whilst CDH1 methylation was associated with a 0.53-fold (95%CI, 0.331–0.844) risk of hrHPV infection persisting over six months of follow-up. In two Dutch cohort studies, FAM19A4/mir124-2 methylation in archived HPV-positive specimens accurately predicted the development of CIN3+ over a follow-up of 14 years [30,31]. Negative methylation results offered better reassurance against cervical cancer than normal cytology results in both studies with a risk difference of 0.71% (95%CI: 0.16–1.40) and 0.98% (95%CI: 0.26–2.00), respectively. However, our data indicated a considerable immediate and long-term risk of CIN2+ among methylation-negative women compared to normal cytology. The difference between these studies might be due to the different study populations, methylation targets, follow-up duration and cytology diagnostic level, etc. Limited by the follow-up duration, we could only evaluate the 24M risk of different methylation results, and a positive methylation result exhibited remarkably higher positive predictive value for CIN2+ both in cross-sectional and longitudinal analysis compared to HPV16/18 and cytology (ASC-US+). This allows us to focus on women at highest risk, but methylation-negative women still need further follow-up due to the insufficiently low cNPV.

Subjective characteristics and complicated logistics of cytology testing were the main obstacles restricting the accuracy of cytology and resulting in significant variation of screening performance between studies. In our study, the sensitivity of cytology in triaging hrHPV-positive women (94%) was much higher than the average level reported from other settings, especially from routine clinical settings. Typically, cytology sensitivity ranges from less than 50% to as high as 90% [31,32,33,34]. This variation is due to the human factor in cytology interpretation. In our study we invited the cytologist from the local hospital for the primary screening and also invited another senior cytology expert from the CHCAMS to review all the slides, resulting in double-reading and therefore a much higher sensitivity of cytology than in most other settings. Unfortunately, it is a great challenge for most LMICs to carry out such high-quality cytology services. In contrast, the sensitivity of HPV16/18 genotyping was comparable between studies, ranging from 50%~65% [35,36], further reinforcing the fact that an objective molecular screening test is more reproducible than one based on morphological interpretations. Compared to cytology, the methylation test has the advantage of being morphology-independent and it can be performed using self-collected samples [37,38]; moreover, compared to HPV16/18 triaging, which is also an objective and suitable method for self-sampling, methylation has the remarkable advantages of detecting more clinically significant lesions, instead of many irrelevant transient HPV infections, thereby preventing unnecessary colposcopy referrals and over-treatment. This fact was reflected by the decreased colposcopy referral rates and increased colposcopy efficiency of methylation triage to detect a CIN2+ case compared to cytology triage and HPV16/18 triage. Of note, thanks to the rapid development of artificial intelligence (AI), the accuracy of cytology is likely to be improved and become more cost-effective [39]. However, more real-world evidence is warranted to confirm its utility in comparison with various biomarker-based screening strategies.

Currently, the greatest challenge in implementation of methylation tests is a lack of consensus on which genes to target for detection. Although there has been very clear evidence on the promising role of methylation biomarkers in cervical cancer screening, the variation in targeted genes, testing methods, evaluated populations and study design makes the evidence fragmentary and heterogeneous. It would be very timely and crucial to evaluate the various genes in the same context with well-designed, prospective, population-based cohort studies in the near future.

The major strength of our study is the evaluation of a panel of methylation assays targeting human gene EPB41L3 and viral regions of HPV16-LI and HPV18-L2 based on a population-based prospective cohort with intensive follow-up. The majority of previous studies on methylation were cross-sectional and conducted among referral population enriched with CIN2+ cases, which did increase the statistical strength of those studies but at the same time might lead to an overestimation of the test performance. Our study more closely represents the real-world performance of a methylation test in a population-based cohort from low-resource settings. The major limitation was the limited number of CIN2+ cases, causing an unstable sensitivity and PPV (wide 95% confidence intervals) of each algorithm, which restricted the statistical power of current analysis; furthermore, the relatively short length of follow-up is another limitation that prevents us from evaluating the most appropriate follow-up intervals for women with negative methylation results. It is therefore important to further evaluate this methylation assay in larger population-based studies with a longer study period and using CIN3+ as the main outcome.

## 5. Conclusions

In summary, DNA methylation targeting the human gene EPB41L3 and viral HPV16L1/HPV18L2 genes could predict the elevated risk of hrHPV persistence and CIN2+. With an increased colposcopy efficiency and specificity with a similar sensitivity, the methylation test could be a promising alternative for HPV16/18 triage and might therefore be considered in strategy of HPV screening and methylation triage followed by immediate treatment of triage-positive women. Although the sensitivity of the methylation test in this study remains suboptimal compared to cytology, in areas that lack well-trained cytology professionals, methylation could be a candidate triage test. However, women with negative methylation results still need further follow-up. Considering the fact that a limited number of CIN2+ in this cohort restricted the statistical strength of the study, further studies with larger populations and longer follow-up periods are strongly encouraged to reinforce the evidence from our study in the near future.

## Figures and Tables

**Table 1:** Methylation positivity by demographic and clinical characteristics (n = 233).

Characteristics	N	Methylation Positivity		*p* Value	OR (95%CI)
		n	Positive Rate		
Age (years)					
20–39	110	6	5.5%	0.002	Reference
≥40	123	23	18.7%		3.99 (1.56–10.20)
Age of menarche (years)					
<12	2	0	/		/
12–17	218	27	12.4%	0.824	Reference
≥18	13	2	15.4%		1.29 (0.27–6.12)
Age of sexual debut (years)					
≤18	33	7	21.2%	0.100	2.18 (0.85–5.60)
>18	200	22	11.0%		Reference
Number of sexual partners					
1	231	29	12.6%	/	/
≥2	2	0	/		
Menopausal status					
Pre-menopausal	157	15	9.6%	0.005	0.15 (0.04–0.52)
Peri-menopausal	12	5	41.7%		Reference
Post-menopausal	64	9	14.1%		0.23 (0.06–0.88)
Syphilis infection					
Yes	11	2	18.2%	0.555	1.60 (0.33–7.82)
No	222	27	12.2%		Reference
hrHPV types					
HPV16/18	57	23	40.4%	<0.001	19.17 (7.26–50.61)
Other hrHPV	176	6	3.4%		Reference
Cytology					
Normal	172	11	6.4%	<0.001 **	Reference
ASC-US/LSIL	41	6	14.6%		2.51 (0.87–7.24)
High grade *	20	12	60.0%		21.95 (7.43–64.86)
Histology outcomes					
No CIN	194	15	7.7%	<0.001 **	Reference
CIN1	22	2	9.1%		1.19 (0.25–5.60)
CIN2	8	5	62.5%		19.89 (4.33–91.41)
CIN3+	9	7	75.0%		41.77 (7.96–219.10)

ASC-US+, atypical squamous cells of undetermined significance or worse; LSIL: low-grade squamous intraepithelial lesions; CIN1-3: cervical intraepithelial neoplasia grade 1–3; OR, odds ratio; CI, confidence interval; * high-grade cytology findings including ASC-US cannot exclude HSIL, atypical glandular cells, HSIL and cancer; ** Chi-square test for trend.

**Table 2:** Cross-sectional accuracy of triage tests for CIN2+ detection among hrHPV-positive women at baseline (n = 233).

Triage Algorithms	Colposcopy Referral Rates (%) (n/N)	Sensitivity (%) (n/N) 95% CI	Specificity (%) (n/N) 95% CI	PPV (%) (n/N) 95% CI	cNPV (%) (n/N) 95% CI	NNR	Relative Sensitivity (95% CI)	Relative Specificity (95% CI)
ASCUS+	26.1 (61/233)	94.1 (16/17) 71.3–99.9	79.2 (171/216) 73.1–84.4	26.2 (16/61) 26.2–15.8	0.6 (1/172) 0–3.2	3.8	Reference 1	Reference 1
LSIL+	15.0 (35/233)	76.5 (13/17) 50.1–93.2	89.8 (194/216) 85.0–93.5	37.1 (13/35) 21.5–55.1	2.0 (4/198) 0.6–5.1	2.7	0.81 (0.64–1.02)	1.13 1.08–1.19
HPV16/18+	24.5 (57/233)	64.7 (11/17) 38.3–85.8	78.7 (170/216) 72.6–84.0	19.3 (11/57) 10.0–31.9	3.4 (6/176) 1.3–7.3	5.2	0.69 (0.49–0.96)	0.99 (0.961–1.09)
Methylation	12.4 (29/233)	70.6 (12/17) 44.0–89.7	92.1 (199/216) 87.7–95.3	41.4 (12/29) 23.5–61.1	2.5 (5/204) 0.8–5.6	2.4	0.75 (0.53–1.06)	1.16 (1.08–1.25)
HPV16/18|ASCUS+	39.9 (93/233)	94.1 (16/17) 71.3–99.9	64.4 (139/216) 57.6–70.7	17.2 (16/93) 10.2–26.4	0.7 (1/140) 0–3.9	5.8	1.0 /	0.81 0.76–0.87
HPV16/18|methylation	27.5 (64/233)	76.5 (13/17) 50.1–93.2	76.9 (166/216) 70.6–82.3	20.6 (13/63) 11.5–32.7	2.4 (4/170) 0.6–6.0	4.8	0.81 0.60–1.10	0.97 0.88–1.07

ASC-US+, atypical squamous cells of undetermined significance or worse; LSIL+: low-grade squamous intraepithelial lesions or worse; CI, confidence interval; CIN2+, cervical intraepithelial neoplasia of grade 2 or worse; PPV, positive predictive value; NPV, negative predictive value, cNPV = 1-NPV; NNR, No. of colposcopies needed to detect per CIN2+ cases.

**Table 3:** Longitudinal accuracy of triage tests among baseline hrHPV-positive women.

Triage Algorithms	Relative Risk of 12M hrHPV Persistence (95% CI)	Relative Risk of 24M hrHPV Persistence (95% CI)	Relative Risk of 24M Total CIN2+ (95% CI)	Sensitivity (%) for 24M Total CIN2+ (n/N) 95% CI	Specificity (%) for 24M Total CIN2+ (n/N) 95% CI	PPV (%) for 24M Total CIN2+ (n/N) 95% CI	cNPV (%) for 24M Total CIN2+ (n/N) 95% CI
ASCUS+	1.27 (0.92–1.74)	1.53 (1.09–2.16)	8.98 (3.81–21.19)	76.0 (19/25) 54.9–90.6	81.5 (132/162) 74.6–87.1	38.8 (19/49) 25.2–53.8	4.3 (6/138) 1.6–9.2
LSIL+	1.29 (0.89–1.86)	1.80 (1.27–2.55)	8.22 (4.10–16.49)	60.0 (15/25) 38.7–78.9	91.4 (148/162) 85.9–95.2	51.7 (15/29) 32.5–70.6	6.4 (10/158) 3.1–12.6)
HPV16/18 positive	1.20 (0.87–1.65)	1.45 (1.02–2.05)	5.49 (2.60–11.57)	64.0 (16/25) 42.5–82.0	81.5 (132/162) 74.6–87.1	34.8 (16/46) 21.4–50.2	6.4 (9/141) 63.0–11.8
Methylation positive	1.89 (1.42–2.52)	1.65 (1.11–2.45)	7.27 (3.68–14.36)	56.0 (14/25) 34.9–75.9	91.4 (148/162) 85.9–95.2	50.0 (14/28) 30.6–69.4	6.9 (11/159) 3.5–12.0
HPV16/18|ASCUS+	1.15 (0.85–156)	1.52 (1.08–2.13)	11.30 (3.51–36.41)	88.0 (22/25) 68.8–97.5	67.9 (110/162) 60.1–75.0	29.7 (22/74) 19.7–41.5	2.7 (3/113) 0.6–7.6
HPV16/18|methylation	1.28 (0.92–1.78)	1.32 (0.90–1.93)	6.91 (3.07–15.55)	72.0 (18/25) 50.6–87.9	79.0 (129/162) 72.6–85.5	35.3 (18/51) 22.4–49.9	5.2 (7/136) 2.1–10.3

ASC-US+, atypical squamous cells of undetermined significance or worse; CI, confidence interval; CIN2+, cervical intraepithelial neoplasia of grade 2 or worse; PPV, positive predictive value; NPV, negative predictive value, cNPV = 1-NPV.

## Data Availability

The datasets used and analyzed during the current study are available from the corresponding author on reasonable request.
